# Exosome ice-needling for chronic wounds in dogs: a case series

**DOI:** 10.3389/fvets.2026.1893100

**Published:** 2026-07-09

**Authors:** Eleonora E. A. Guidi, Kyung Min Lim, Alessandro Negro, Antonella Vercelli, Luisa Cornegliani

**Affiliations:** 1 Clinica Veterinaria Città di Torino, Turin, Italy; 2 StemExOne Co., R&D Team, Seoul, Republic of Korea

**Keywords:** chronic wounds, dog, exosomes, ice-needling, plant-derived exosome-like nanovesicles, regenerative medicine, wound healing

## Abstract

Chronic wounds in dogs represent a significant clinical challenge due to persistent inflammation and impaired tissue repair. This case series describes four canine patients with chronic, treatment-resistant cutaneous wounds treated with plant-derived exosome-like nanovesicles (PNELs) delivered via ice-needling technology (IN). Three dogs presented with post-surgical wound dehiscence and one dog with a chronic pressure sore. The dogs included in this case series presented with non-healing cutaneous lesions characterized by persistent wound dehiscence or pressure ulceration despite prior surgical and medical management. Clinical examination revealed chronic inflammation, impaired granulation tissue formation, and delayed epithelialization. All cases had previously failed conventional therapies, including surgical revision, topical management, systemic treatments, and photobiomodulation. Weekly PNEL applications were performed using IN without additional wound-specific interventions. All dogs demonstrated rapid clinical improvement characterized by reduced inflammation, healthy granulation tissue formation, progressive wound contraction, and accelerated epithelialization. Complete wound healing was achieved within 2 to 12 weeks depending on lesion chronicity and severity. No local or systemic adverse effects were observed. These preliminary findings suggest that PNEL ice-needling may have potential as a minimally invasive regenerative approach for chronic wound management in veterinary patients. Further controlled clinical studies are warranted to confirm efficacy and safety and to establish standardized treatment protocols.

## 1 Introduction

Skin wound healing is a complex and highly coordinated biological process involving inflammatory regulation, cell migration and proliferation, extracellular matrix remodeling, and angiogenesis. Chronic wounds fail to progress through the normal stages of healing because of persistent inflammation, impaired neovascularization, and dysregulated cellular responses (1). These lesions are characterized by reduced levels of growth factors, excessive protease activity, bacterial contamination, and reduced oxygen and nutrient supply, ultimately resulting in delayed or incomplete healing (1, 2).

Conventional management strategies for chronic wounds in veterinary medicine include surgical revision, topical therapies, physical modalities such as photobiomodulation, and pharmacological interventions (3, 4). However, these approaches are frequently insufficient in chronic or treatment-resistant lesions, highlighting the need for regenerative therapies capable of actively promoting physiological repair mechanisms (4, 5).

Cell-free regenerative therapies have emerged as promising treatment options because of their low immunogenicity, ease of production and handling, and long-term storage stability (5). Exosomes are nanosized extracellular vesicles released by cells into the extracellular environment where they mediate intercellular communication and regulate multiple biological processes (6). Animal-derived exosomes have demonstrated anti-inflammatory, pro-angiogenic, and regenerative effects in wound healing. Nevertheless, they may trigger immune responses, are difficult to isolate, and are susceptible to degradation (7).

Plant-derived exosome-like nanovesicles (PNELs) have recently gained attention as potential alternatives to animal-derived vesicles. PNELs possess favorable safety profiles, enhanced biological stability, and intrinsic regenerative properties (3, 7). Their bioactive cargo, including lipids, proteins, antioxidant molecules, and regulatory RNAs, promotes cross-kingdom signaling capable of modulating inflammation, stimulating fibroblast and keratinocyte proliferation, enhancing angiogenesis, and accelerating extracellular matrix deposition (3). Some PNELs have also demonstrated antibacterial activity (3). These intrinsic regenerative and anti-inflammatory properties of PNELs parallel the well-documented effects of mammalian MSC-derived exosomes (8, 9). Furthermore, their antioxidant capacity shares functional similarities with established immunomodulatory agents like lipoic acid (10).

Efficient delivery of exosomes to target tissues remains a major challenge. Ice-needling technology is a novel transdermal delivery system in which therapeutic solutions are converted into micro-ice particles capable of penetrating the skin surface and releasing bioactive compounds uniformly into target tissues while minimizing product loss (11). Preliminary experimental studies have reported reduced inflammation and enhanced tissue repair following exosome delivery using ice-needling technology; however, evidence remains limited (11, 12).

The aim of this case series is to describe the clinical outcomes of four dogs with chronic, treatment-resistant wounds treated with PNELs delivered through IN.

## 2 Materials and methods

### 2.1 Study design

This study is a retrospective case series involving four client-owned dogs presenting with chronic, non-healing cutaneous wounds.

### 2.2 Inclusion criteria

Cases were included based on the following criteria:

- Wound duration greater than 4 weeks
- Failure of previous conventional treatments
- Availability of complete clinical follow-up until healing

### 2.3 Treatment protocol

All dogs were treated with plant-derived exosome-like nanovesicles (VetEase®, RecensMedical Inc., South Korea) from *Houttuynia Cordata*, *Selaginella Tamariscina* and *Centella Asiatica* delivered via IN. Treatments were performed once weekly. No additional topical or systemic wound-specific therapies were administered during the treatment period.

### 2.4 Outcome assessment

Clinical outcomes were evaluated based on:

- Reduction in inflammation
- Development of granulation tissue
- Wound contraction
- Time to complete epithelialization
- Occurrence of adverse effects

### 2.5 Ethical considerations

Ethical review and approval were waived given the retrospective nature of the study, and Good Clinical Practice (VICH GL9) principles were respected. The product used in this study is intended for cosmetic use and, as a plant-derived product, is not subject to regulations applicable to veterinary medicinal drugs. Informed consent was obtained from all owners.

## 3 Results

### 3.1 Case descriptions

#### 3.1.1 Case 1

**Figure 1:**
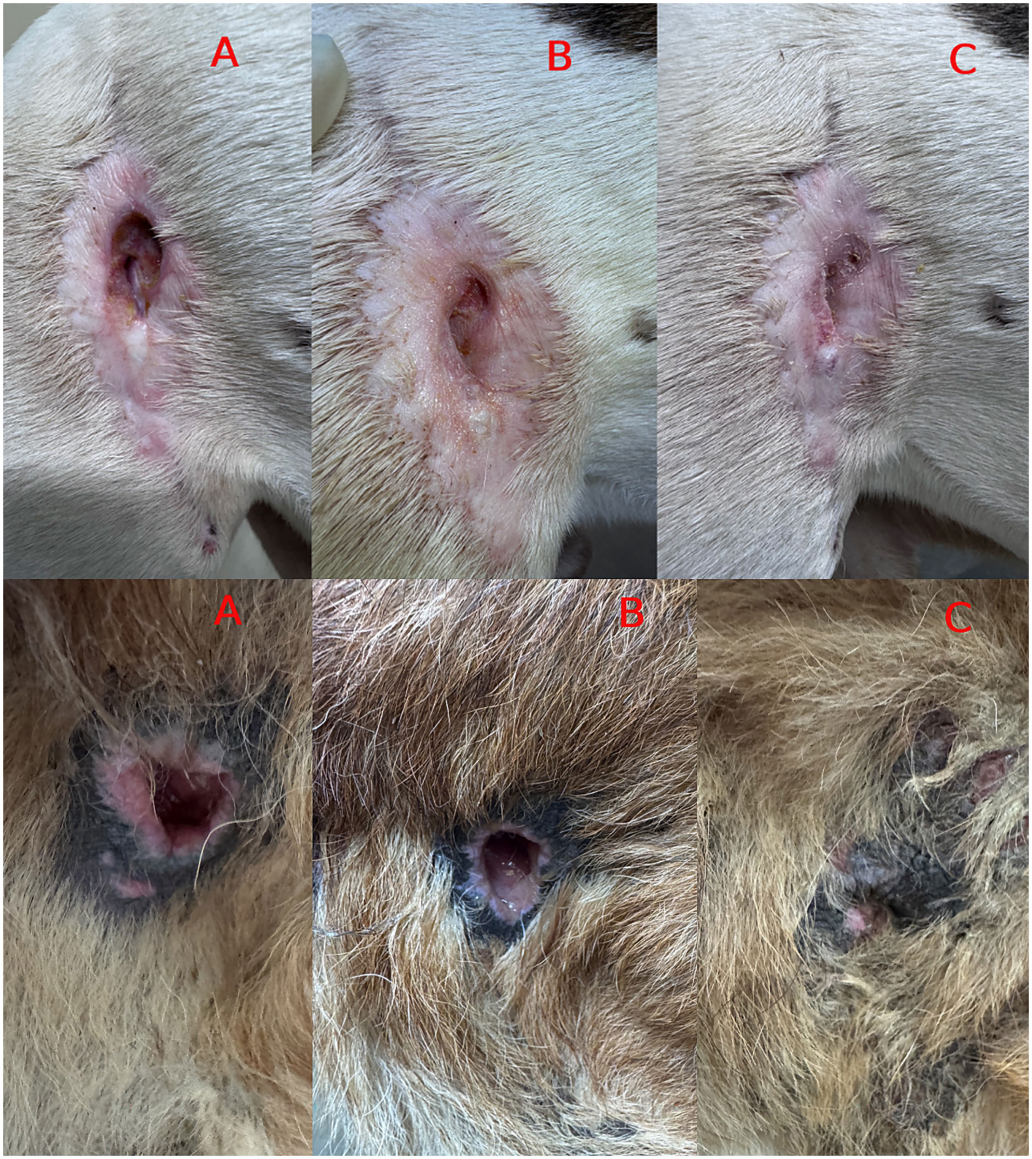
Upper line: Case 1 evolution: **(A)** day 0, **(B)** day 21, **(C)** day 42. Bottom line: Case 2 evolution: **(A)** day 0, **(B)** day 45, **(C)** day 90.

An 8-year-old male Pitbull underwent surgical excision of a cutaneous mast cell tumor with regional lymph node removal. Two weeks postoperatively, wound dehiscence developed. Despite surgical revision, topical therapy, and photobiomodulation, the lesion persisted for 6 months. Weekly PNEL ice-needling (VetEase®, RecensMedical Inc., South Korea) therapy was initiated. Within 2 weeks, a reduction in inflammation and early granulation tissue formation were observed. By weeks 3–4, marked wound contraction and improved tissue organization were evident. Complete epithelialization was achieved by week 6 (Figure 1).

#### 3.1.2 Case 2

A 9-year-old male German Shepherd dog was referred for a pressure sore on the right hip present for approximately 1 year. Previous topical and systemic treatments were ineffective. Weekly PNEL ice-needling (VetEase®, RecensMedical Inc., South Korea) therapy was administered without concomitant medications. Progressive reduction in lesion size and improved granulation tissue formation were observed at 15 and 30 days. Continued treatment resulted in gradual epithelialization and complete closure by day 90 (Figure 1).

#### 3.1.3 Case 3

**Figure 2:**
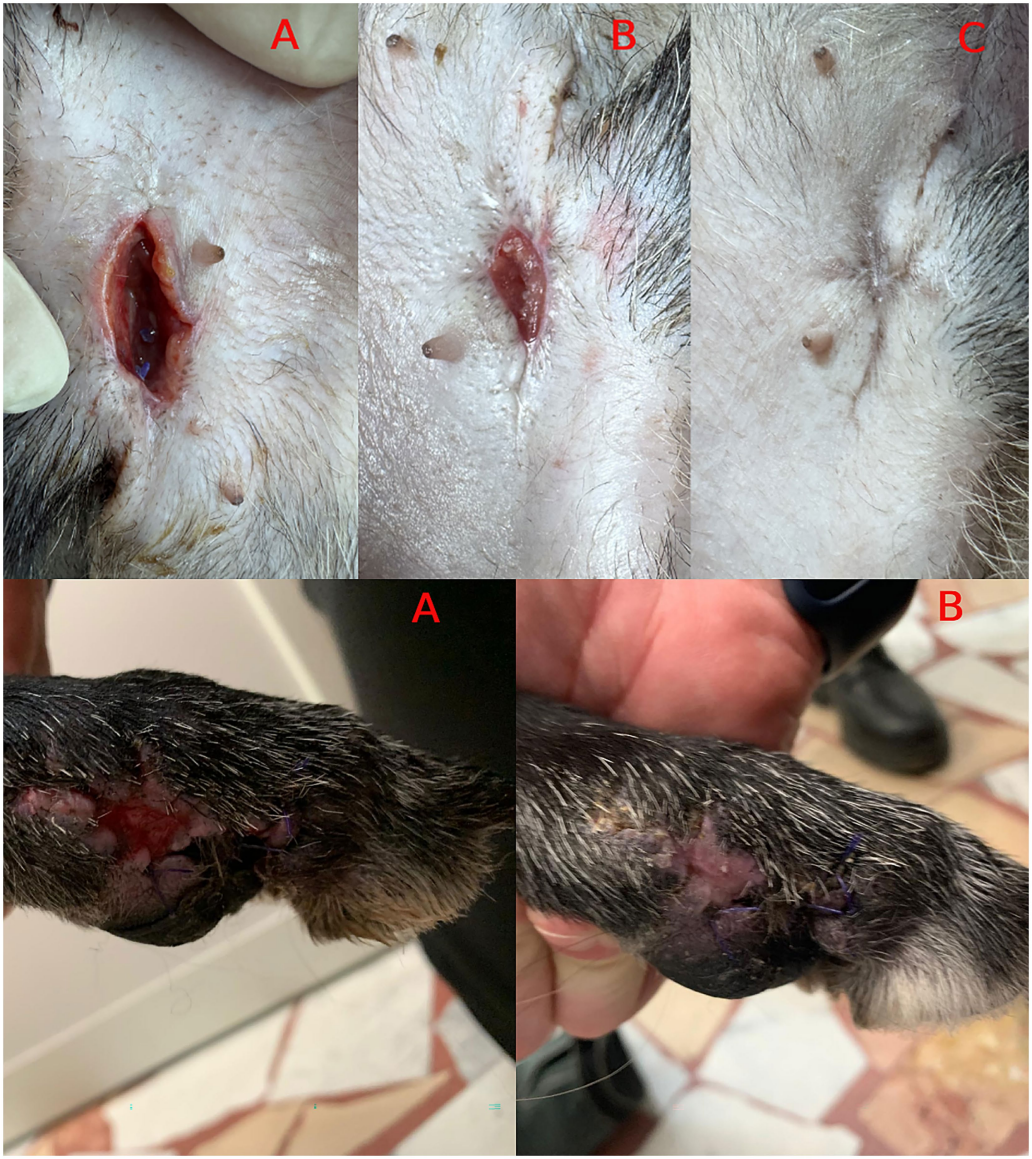
Upper line: Case 3 evolution: **(A)** day 0, **(B)** day 7, **(C)** day 14. Bottom line: Case 4 evolution: **(A)** day 0, **(B)** day 14.

A 2-year-old male mixed-breed dog underwent cystotomy for bladder stone removal. Postoperative wound dehiscence after 10 days prompted surgical re-intervention, but dehiscence recurred after 1 week. Following unsuccessful topical therapy, weekly PNEL ice-needling (VetEase®, RecensMedical Inc., South Korea) treatment was started. Rapid granulation tissue formation and wound contraction were noted after the first session. Complete epithelialization occurred within 15 days, requiring only two treatments (Figure 2).

#### 3.1.4 Case 4

A 9-year-old spayed female mixed-breed dog presented with wound dehiscence 2 weeks after digit amputation for subungual squamous cell carcinoma. Despite 4 weeks of topical management, healing was minimal. Weekly PNEL ice-needling (VetEase®, RecensMedical Inc., South Korea) therapy was initiated, resulting in complete wound closure within 2 weeks (Figure 2).

### 3.2 Clinical outcomes

All four dogs demonstrated progressive wound healing following initiation of treatment. Reduction in local inflammation was observed within 1–2 weeks, followed by healthy granulation tissue formation, wound contraction, and epithelialization. Complete wound closure was achieved in all dogs. Time to complete healing ranged from 2 to 12 weeks depending on wound chronicity and size.

No local or systemic adverse effects, delayed reactions, or signs of intolerance were observed during treatment or follow-up in these cases.

Table 1 summarizes patient characteristics, previous treatments, and clinical outcomes.

**Table 1:** Summary of patient characteristics, previous treatments, and clinical outcomes.

Case	Patient	Condition	Previous treatments	Outcome of previous treatments	Subsequent intervention	Final outcome
Case 1	Pitbull, 8 y/o, M	Post-surgical wound dehiscence (mast cell tumor excision)	Surgical revision, topical therapy, photobiomodulation	Ineffective; lesion persisted for 6 months	Weekly PNEL ice-needling	Complete epithelialization in 6 weeks
Case 2	German Shepherd, 9 y/o, M	Chronic pressure sore (right hip, 1 year)	Topical and systemic therapies	Ineffective	Weekly PNEL ice-needling	Complete closure in 90 days
Case 3	Mixed-breed, 2 y/o, M	Post-cystotomy wound dehiscence	Surgical re-closure and topical therapy	Recurrence after 1 week; ineffective	Weekly PNEL ice-needling	Complete epithelialization in approximately 15 days
Case 4	Mixed-breed, 9 y/o, spayed F	Post-amputation wound dehiscence (subungual squamous cell carcinoma)	Four weeks of topical management	Minimal healing	Weekly PNEL ice-needling	Complete closure in 2 weeks

## 4 Discussion

This case series provides preliminary clinical outcomes following treatment with PNELs delivered via IN for the management of chronic wounds in dogs.

Chronic wounds are characterized by prolonged inflammation, impaired angiogenesis, excessive protease activity, and defective cellular responses, all of which contribute to delayed healing and increased risk of complications (1, 2). Regenerative approaches aimed at restoring physiological healing mechanisms are therefore gaining increasing attention in both veterinary and human medicine (4, 5).

Exosome-based therapies have demonstrated significant regenerative potential through modulation of macrophage polarization, stimulation of fibroblast proliferation, enhancement of keratinocyte migration, promotion of angiogenesis, and extracellular matrix remodeling (13, 14). These mechanisms collectively accelerate wound closure and improve tissue quality. The clinical improvements observed in this study, particularly rapid granulation tissue formation and epithelialization, are consistent with these proposed biological effects.

Plant-derived exosome-like nanovesicles may offer several advantages over animal-derived exosomes, including lower immunogenicity, greater structural stability, and unique cross-kingdom signaling properties (3, 7). Their lipid composition and bioactive molecular cargo facilitate modulation of inflammatory pathways while promoting tissue repair and angiogenesis (3). In addition, the robust lipid bilayer of PNELs may enhance resistance to physical stress and cryogenic conditions, potentially preserving biological activity during ice-needling delivery. Despite these promising properties, the broad clinical translation of PNELs is currently limited by technical challenges, including achieving yield consistency, standardizing isolation protocols to ensure high purity, and the need for comprehensive *in vivo* mechanistic validation (15, 16).

The delivery method may have contributed to the observed outcomes, although its specific role cannot be determined from the present case series. IN enables uniform distribution and efficient transdermal penetration of therapeutic compounds without the invasiveness associated with repeated injections (11). Reduced product loss and improved local bioavailability may contribute to the rapid tissue responses observed in these cases.

From a clinical perspective, the minimally invasive nature of IN is particularly advantageous in veterinary patients, where repeated injections or extensive wound manipulation may be poorly tolerated. Treatments in the present series were rapidly performed and did not require sedation.

Importantly, no adverse effects, delayed reactions, or signs of intolerance were observed. This finding is consistent with previous reports describing the favorable safety and biocompatibility profile of PNELs and exosome-based therapies (3, 12).

This study has several limitations, including the small sample size, lack of a control group, heterogeneity of wound types, and absence of standardized wound scoring or histological evaluation. These limitations prevent definitive conclusions regarding efficacy and treatment superiority over conventional therapies. While no adverse effects were observed in this short-term case series, we acknowledge that larger, controlled studies are required to establish the long-term biosafety and clinical standardization of PNELs (17).

Nevertheless, the consistent clinical improvement observed across these cases suggests that PNEL ice-needling therapy merits further investigation as a potential adjunctive approach for veterinary wound management. Further controlled clinical studies are required to validate these findings, optimize treatment intervals, and compare outcomes with established therapeutic approaches.

In this case series PNEL’s delivered via IN achieved healing in chronic, complex, and treatment-resistant wounds. This minimally invasive approach was well tolerated and associated with rapid granulation tissue formation, wound contraction, and epithelialization. These preliminary findings support further investigation through controlled clinical studies to confirm efficacy and establish standardized treatment guidelines.

## Data Availability

The raw data supporting the conclusions of this article will be made available by the authors, without undue reservation.
